# Predictive value of the presence of *Prevotella* and the ratio of *Porphyromonas gingivalis* to *Prevotella* in saliva for esophageal squamous cell carcinoma

**DOI:** 10.3389/fcimb.2022.997333

**Published:** 2022-10-13

**Authors:** Xiaohui Chen, Bohong Xian, Junmin Wei, Yixiang Chen, Dongyang Yang, Xiaorong Lai, Lifang Liu, Yinghong Wu, Xiayi Lin, Yu Deng, Huabin Zhang, Wanwei Liu, Guibin Qiao, Zijun Li

**Affiliations:** ^1^ Department of General Practice, Guangdong Provincial People's Hospital, Guangdong Academy of Medical Sciences, Guangzhou, China; ^2^ Department of General Practice, Guangdong Provincial People's Hospital, Concord Medical Center, Guangdong Academy of Medical Sciences, Guangzhou, China; ^3^ Department of Gastroenterology, Guangdong General Hospital, Guangdong Academy of Medical Sciences, Guangzhou, China; ^4^ Medicine-Oncology, Guangdong Provincial People’s Hospital, Guangdong Academy of Medical Sciences, Guangzhou, China; ^5^ Guangdong Provincial Geriatrics Institute, Guangdong Provincial People's Hospital, Guangdong Academy of Medical Sciences, Guangzhou, China; ^6^ Department of Thoracic Surgery, Guangdong Provincial People’s Hospital, Guangdong Academy of Medical Sciences, Guangzhou, China

**Keywords:** esophageal squamous cell carcinoma (ESCC), biomarker, salivary bacteria, diagnostic performance, receiver operating characteristic (ROC)

## Abstract

**Background:**

Imbalance of oral salivary microbiota has been linked to the pathogenesis of a variety of systemic diseases, and oral bacterial species have been shown to be useful biomarkers for systemic diseases.This study aimed to characterize the alterations of oral microbiota in patients with esophageal squamous cell carcinoma (ESCC) and to evaluate the diagnostic performance of oral microbial biomarkers for ESCC.

**Methods:**

The relative abundance of flora in saliva samples was analyzed by 16S rDNA sequencing, and differences in the species present in samples from ESCC patients and healthy controls (HCs) were identified by analyzing species diversity and performing LEfSe analysis. Receiver operating characteristic (ROC) curve analysis was applied to evaluate the diagnostic performance of the characteristic bacteria individually and in combination.

**Results:**

Differences in bacterial diversity indexes were observed for the saliva of ESCC patients versus HCs (*P*<0.05), but principal coordinate analysis did not detect a significant difference in the composition of oral microbiota between ESCC patients and HCs (*P*>0.05). LEfSe analysis showed that *Leptotrichia*, *Porphyromonas (Pg)*, *Streptococcus*, *Rothia*, *Lactobacillus* and *Peptostreptococcus* were more abundant in ESCC patient saliva than in HC saliva, whereas *Haemophilus, Alloprevotella* (*All*), *Prevotella_7, Prevotella* (*Pre*), *Prevotella_6*, *Pasteurellaceae* and *Pasteurellales* were significantly less abundant in ESCC patient saliva (*P*<0.05). From ROC curve analysis, *Pg* could detect ESCC with an area under the ROC curve (AUC) of 0.599, sensitivity of 62.2%, and specificity of 70%, whereas the ratio of *Pg/Pre* had an AUC of 0.791, sensitivity of 93.3%, and specificity of 62.3%. Moreover, the combination of the *Pg/Pre* and *Pg/All* ratios showed further improved diagnostic performance for ESCC (AUC=0.826) and even good sensitivity and specificity for the diagnosis of early ESCC (68.2% and 86%, respectively; AUC=0.786).

**Conclusion:**

This study shows that *Pg* in saliva can be used as a characteristic marker of ESCC, and the ratios of *Pg/Pre* and *Pg/All* offered significantly improved diagnostic performance, especially for early ESCC.

## Introduction

Esophageal cancer is a common malignant tumor type and ranks 7th in terms of cancer morbidity (Sung et al., 2021). Approximately 50% of cases worldwide occur in China, and 90% of these cases are esophageal squamous cell carcinoma (ESCC) (Li et al., 2021). Because ESCC commonly produces only mild symptoms in early stages, most cases are not diagnosed until the disease ha progressed to advanced stages, at which time treatment effectiveness is poor, resulting in a low 5-year survival rate (Roshandel et al., 2013). Accordingly, the identification of characteristic markers of early ESCC that facilitate early diagnosis is very important for improving the clinical prognosis and quality of life of ESCC patients. To date, no molecular markers have been established for accurate detection of ESCC in clinical practice. Instead, tissue and blood samples are required to detect and diagnose esophageal cancer, and due to the invasiveness of their collection, such testing is difficult to popularize for regular screening. As an alternative, human saliva is an attractive clinical diagnostic medium that can be easily and non-invasively collected.Considerable research in recent decades has sought to identify saliva biomarkers for a variety of diseases (Sugimoto, 2020), and studies have shown that saliva components may be useful as diagnostic markers for oral cancer (Li et al., 2004), Sjogren’s syndrome (Tandon et al., 2012), breast cancer (Zhang et al., 2010b), pancreatic cancer (Zhang et al., 2010a) and lung cancer (Zhang et al., 2012). However, few studies have investigated saliva-related markers for esophageal cancer.

The oral microbiota is the second largest after that of the intestinal tract, containing more than 700 species of bacteria along with fungi, viruses and protozoa (Caselli et al., 2020). Abnormal oral mircrobiota is closely related to the occurrence of many diseases. Recent studies has shown that dysbiosis of oral microbiota may contribute to ESCC (Narikiyo et al., 2004; Chen et al., 2015; Michaud et al., 2016; Peters et al., 2017; Snider et al., 2018; Wei et al., 2022). Furthermore, retrospective case-control and large-scale prospective cohort studies (Abnet et al., 2001; Guha et al., 2007; Abnet et al., 2008; Zhang et al., 2019; Yano et al., 2021b) have demonstrated that the structure of oral flora in ESCC patients are significantly different from that in normal individuals and there is a close association between the alteration of the oral microbiome and the development of ESCC (Yano et al., 2021a), indicating a pathogenetic role of oral microbiota in ESCC. However, the specific oral bacteria that are responsible for the close association or the pathogenetic role remain to be determined. Identification of the responsible bacteria and understanding of their role in the etiology of ESCC are particularly important for further development of convenient early diagnostic methods as well as strategies for the prevention of ESCC. Accordingly, characterization of salivary microbiota associated with ESCC is essential.

Furthermore, we believe that a variety of bacteria and microorganisms interact with each other and play a role in the occurrence and development of ESCC. Several experimental studies have suggested that flora imbalance promotes the development of ESCC. Liu et al. (Liu et al., 2018) found that an increased combined abundance of *Streptococcus* and *Prevotella* was related to the prognosis of ESCC patients. Another study by Yang et al. showed that ESCC-specific microbial taxa may serve as sensitive and specific clinical diagnostic markers (Yang et al., 2021). However, these studies did not identify a specific index for the prediction of ESCC.

In the present study, we aimed to identify microbiota associated with ESCC as well as the relationships among microbiota that may provide useful biomarkers for early ESCC detection. Based on the method used by Guo et al. (Guo et al., 2018), we considered the ratios of different flora and analyzed their diagnostic value for ESCC. Using 16s ribosomal RNA (rRNA) sequencing of saliva samples, we directly compared the composition of salivary microflora between newly diagnosed and untreated ESCC patients and healthy controls, and then selected microflora that showed large differences between these groups for ratio analysis.

## Materials and methods

### Study population

Patients with ESCC who underwent thoracoscopic partial esophagectomy or gastroscopy in Guangdong Provincial People’s Hospital between June 2018 and July 2020 were screened according to the following inclusion criteria: (1) age >18 years; (2) histopathological diagnosis of ESCC, (3) good overall health, with no metabolic diseases such as diabetes or hyperlipidemia and no infectious diseases; (3) no use of antibiotics, acid suppressants, or probiotics that affect esophageal flora in the previous 2 months; (4) no special eating habits, such as chewing betel nut; and (5) the absence of severe liver and renal insufficiency or immune deficiency. Patients were further excluded from this study if they: (1) had taken drugs that affect the esophageal microecology within the previous 2 months; (2) had an autoimmune disease; (3) previously or currently had tumors other than esophageal cancer; and (4) had incomplete clinical data. Saliva samples were collected from the included ESCC patients. Fifty healthy individuals for whom no abnormal results were found on gastroscopy in Guangdong Provincial People’s Hospital in the same period were included as healthy controls (all healthy individuals had a body mass index within the range of normal for them). The study protocol was examined and approved by the Ethics Committee of Guangdong Provincial People’s Hospital, and all participants provided written informed consent.

### Saliva sample collection

For the collection of naturally secreted saliva samples, participants gargled with normal saline to remove food residue and then did not speak, eat, or drink water for at least 5 min before sample collection. Each saliva sample of no less than 3 ml was spilt into a saliva collection tube and immediately stored at –80°C until later extraction of DNA.

### DNA extraction and sequencing

A suspension of 0.5 ml saliva and 1 ml phosphate-buffered saline (PBS) buffer (pH 7.4) was centrifuged at 3000*g* for 5 minutes, and the supernatant was discarded. After the precipitant was resuspended with 200 μl PBS and 20 μl protease, DNA was extracted from saliva samples using the UltraClean^®^microbial DNA separation kit (MOBI, USA). The concentration and purity were measured using the NanoDrop One (Thermo Fisher Scientific, MA, USA). The filtrate was stored at –20°C. With this kit, if the total DNA mass is ≥150 ng and the DNA concentration is ≥5 ng/μl, the main band will be obvious and not appear degraded or contaminated by DNA or protein.

### 16s rRNA sequencing data processing

The V4 region of bacterial and archaeal 16S rRNA genes was amplified with prokaryotic universal primers F515 (5’-GTGCCAGCMGCCGCGGTAA-3’)and R806 (5’-GGACTACVSGGGTATCTAAT-3’) (Bates et al., 2011) and a sample-specific 12-bp barcode on F515. The amplification process included an initialization step at 94°C for 5 min followed by 30 denaturation cycles at 94°C for 30 sec, an annealing step at 52°C for 30 sec, an extension step at 72°C for 30 sec, and a final extension step at 72°C for 10 min. The fragment lengths and concentrations of the PCR products were detected by 1% agarose gel electrophoresis (Guangzhou HaoMa Biotechnology Co., Ltd, Guangzhou, China), and the product concentrations were compared and analyzed using GeneTools analysis software (Version 4.03.05.0, SynGene,USA). The mixed PCR product was purified using the EZNA gel extraction kit (Omega, USA) and eluted with TE buffer (REGAL, Shanghai, China) to recover the target DNA fragments. A DNA library was established using the DNA library preparation toolkit of Nebnext Ultra standard program specification for Illumina^®^ (New England Biolabs, USA). The DNA amplification sublibrary was sequenced using the PE250 paired-end reads on the Illumina Hiseq2500 platform, and the paired terminal original readings were screened by the Trimmomatic software (V0.33, USADELLAB.org). Each paired end (PE) was read by FLASH software (Fast Length Adjustment of SHort reads, V1.2.11, https://ccb.jhu.edu/software/FLASH/), and the original tag sequence was filtered by Mothur software (V1.35.1, http://www.mothur.org). Finally, a clean tag was obtained.

### Bioinformatics analysis

Using UPARSE software (Version 10, http://www.drive5.com/usearch/), the cutoff value for identification of a sequence is 97%. The most frequently read operational taxonomic unit (OTU) representative sequences were assigned to annotated species at different classification levels, and then the size of the linear discriminant analysis (LDA) effect (LDA Effect Size tool, LEfSe) (Segata et al., 2011) was analyzed using the QIIME-based Ribosomal Database Project (RDP) classifier wrapper (Caporaso et al., 2010). LEfSe analysis is used to discover high-dimensional biomarkers and reveal genomic characteristics. It achieves comparative analysis among multiple groups for the purpose of identifying species present at significantly different abundances among groups.

The bacterial diversity and richness or abundance in the samples were evaluated by calculating the following parameters or indices: Chao1, ACE, Simpson,and Shannon, with an estimated distance of 3%. Weighted and unweighted UniFrac indexes were used to evaluate the diversity of salivary microbiota in the ESCC and HC groups. The UniFrac distance is based on the branch length fraction shared between two communities in the phylogenetic tree constructed from the 16SrRNA gene sequences of all comparative communities. LEfSe analysis was used to identify microbial groups with biomarker characteristics at multiple levels in saliva samples, to classify them quantitatively according to statistical significance, and to produce classification bar charts and branching graphs for visualization of the results.

### Statistical analysis

SPSS 21.0 software (SPSS, Inc., USA) was used for all statistical analyses. Measurement data that followed a normal distribution were expressed as mean ± standard deviation (SD) and compared using an independent sample t-test.The microflora abundance values were expressed as percentages and compared with nonparametric Kruskal–Wallis test and Wilcoxon rank sum test. Biomarker performance for the diagnosis of ECSS was analyzed by receiver operating characteristic (ROC) curve analysis. Values of *P*<0.05 were considered to indicate a statistically significant difference.

## Results

### Baseline characteristics of ESCC patients and healthy controls

A total of 90 patients newly diagnosed with ESCC were included in this study. These patients included 71 men and 19 women, with a mean age of 60.8 ± 7.49 years. The 50 healthy controls included 27 men and 23 women, with a mean age of 47.7 ± 13.8 years (**Table 1**). Among the 90 ESCC patients, pathology and staging (TNM staging) were determined for 53 patients and showed that 21 patients had early-stage ESCC (stage I + II) and 32 patients had late-stage ESCC (III + IV).

**Table 1:** Clinical information of patients in the ESCC and healthy control (HC) groups.

Baseline characteristics		ESCC (n=90)	HC (n=50)	*P*
**Age (year, mean ± SD)**		60.8 ± 7.49	47.7 ± 13.8	<0.001
**Sex**	Male	71	27	<0.01
	Female	19	23	
**Smoking**	Yes	52	12	<0.01
	No	38	38	
**Drinking**	Yes	50	6	<0.01
	No	40	44	
**ESCC family history**	Yes	6	1	0.225
	No	84	49	
**Daily teeth brushing times**	<2	68	41	0.379
	>2	22	9	

P-values were determined by Wilcoxon rank sum test for continuous variables and chi square test for categorical variables (bilateral).

### Diversity of salivary microbiota in patients with ESCC and in healthy individuals

**Figure 1:**
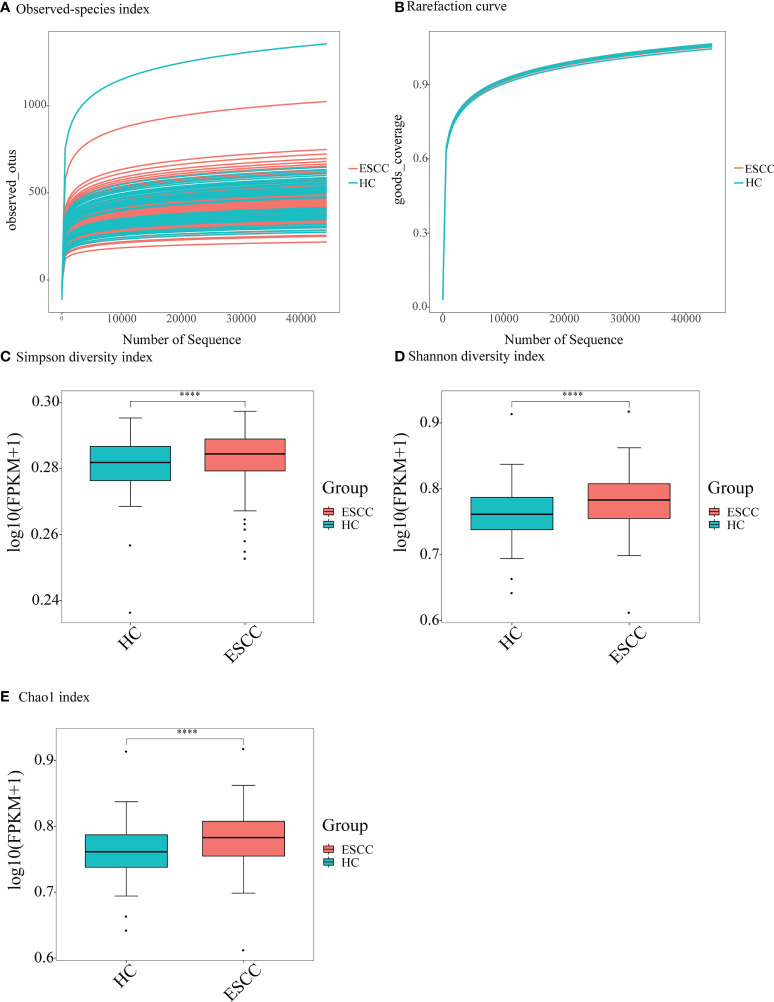
Alpha-diversity analysis showing difference in the abundance and diversity of saliva microbiota in the ESCC and healthy control (HC) groups. **(A)** Observed-species index; **(B)** rarefaction curve; **(C)** Simpson diversity index, **(D)** Shannon diversity index, and **(E)** Chao1 index. ****P < 0.00001.

After sequence denoising and pruning as well as chimera selection, a total of 14,654,071 taxon readings were analyzed. The readings clustered into 103,003 OTUs at the 97% similarity threshold level, including 65,664 OTUs in the ESCC group and 37,339 OTUs in the healthy control group. We first checked the sequencing depth by generating a sparse curve for each sample (**Figure 1A**). Most samples reached the platform stage, indicating that the sequencing was sufficient. In addition, the coverage exceeded 0.99 for both groups, indicating that the readings obtained from both groups represented most of the bacteria present (**Figure 1B**). Next, we compared the microbial diversity and richness between the groups according to the Chao1, Shannon, and Simpson indexes. The Chao1 abundance diversity index for the ESCC group was significantly lower than that for the control group (**Figure 1E**), whereas the Simpson and Shannon diversity indexes for the ESCC group were significantly higher than those for the control group (**Figures 1C, D**).

**Figure 2:**
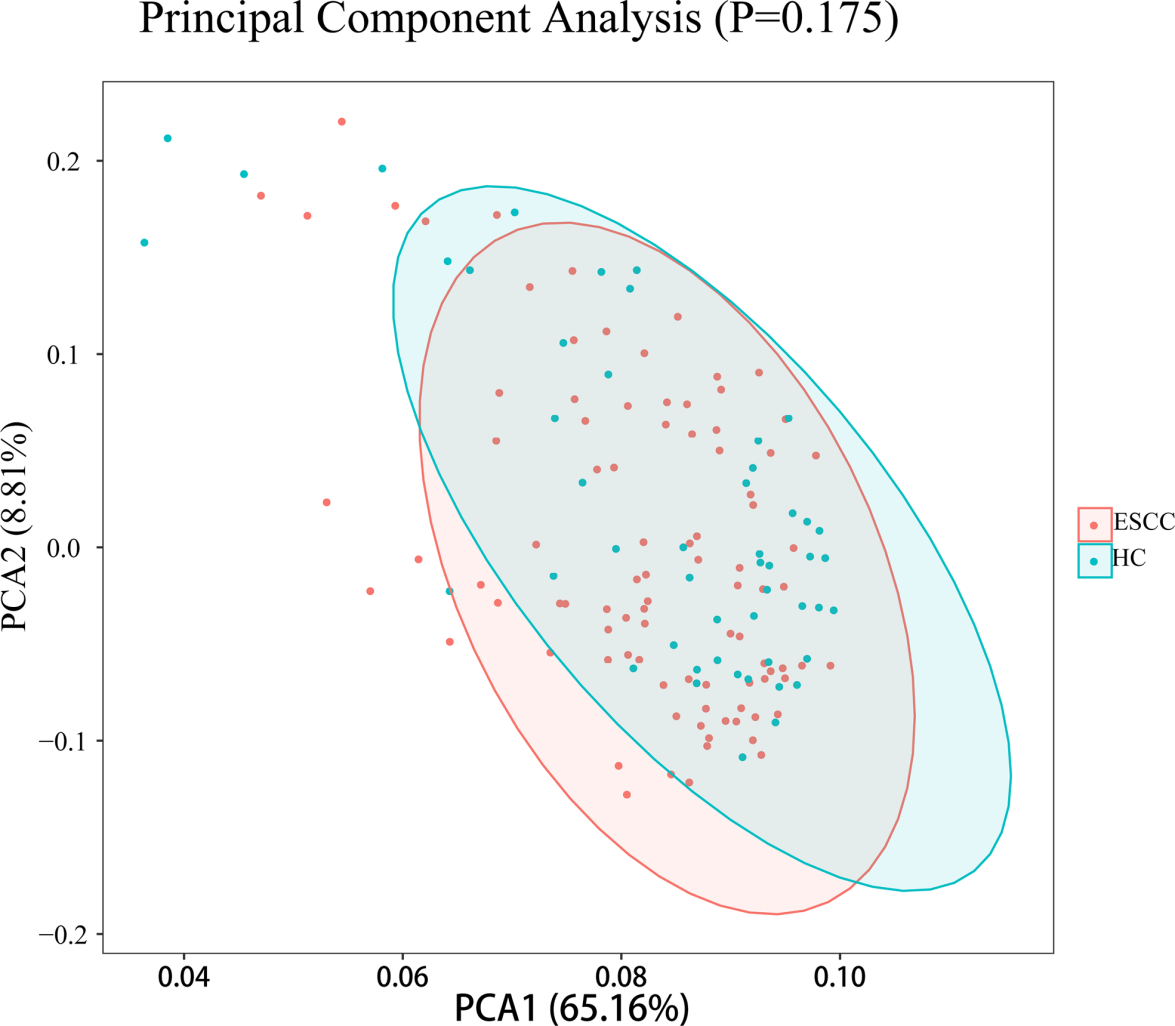
PCoA plots for saliva samples from the ESCC and healthy control (HC) groups.

To test whether the diversity of salivary microbiota can distinguish the ESCC group from the healthy control group, PCoA was performed to compare the overall structure of the microbial communities in the saliva samples of the two groups. PCoA showed no significant differences in the overall composition of the microecology between the two groups (**Figure 2**).

### Comparison of salivary microbiota composition between ESCC patients and healthy controls

**Figure 3:**
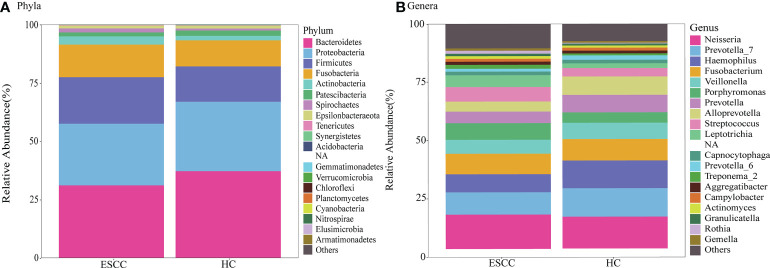
Taxonomic composition of saliva microbiota of ESCC patients and healthy controls. Relative abundance bar plots show the relative abundance of bacterial **(A)** phyla and **(B)** genera in each sample.

We next compared the relative abundances of microbiota between ESCC patients and healthy controls. More than 200 genera were classified within the salivary microbiota. The main bacterial composition at the phylum level (total sequences>1% in any group) included Bacteroidetes, Proteobacteria, Firmicutes, Fusobacteria, Actinobacteria, Patescibacteria, Spirochaetes and Epsilonbacteraeota, accounting for 99.53% and 99.54% of salivary microbiota in saliva samples from ESCC patients and healthy controls, respectively (**Figure 3A**). The dominant genera were similar between the ESCC and healthy control saliva samples. *Neisseria* was the most abundant genus in both the ESCC group and healthy control group, and *Prevotella 7* was the second most abundant genus (**Figure 3B**).

Next, we performed LEfSe analysis to determine the maximum differences in classification groups between the ESCC and healthy control groups. We found that Bacilli and Firmicutes levels were significantly increased in the ESCC group compared with the healthy control group, and *Leptotrichia* and *Porphyromonas (Pg)* showed the greatest increases in the ESCC group compared with the healthy control group at the genus level. Saliva samples of the ESCC group also contained more *Streptococcus*, *Rothia*, *Lactobacillus* and *Peptostreptococcus*. In saliva samples from the healthy control group, Bacteroidetes was the most abundant phylum, and *Haemophilus*, *Alloprevotella (All)*, *Prevotella 7*, and *Prevotella (Pre)* were the most prominent genera. Other genera found at elevated levels in the healthy control group included *Prevotella 6*, *Pasteurellaceae*, and *Pasteurellales*.

### Evaluation of indexes of salivary flora imbalance (Pg/All and Pg/Pre) in ESCC patients

From the findings presented above and the work of others, we know there is significant alteration in the abundance of some flora in the saliva of ESCC patients. However, increasing evidence indicates that combinations of bacterial genera altered in disease conditions offer improved diagnostic ability compared with individual genera. Guo et al. (Guo et al., 2018) demonstrated that the microbial ratio detection method is conducive to improving the diagnostic ability of multiple bacterial genera in disease.Therefore, according to previous research, we selected the flora *Pg*, which showed an increased relative abundance in ESCC patients and has been potentially linked to the development of ESCC (Wei et al., 2022), as well as the flora *All* and *Pre*, which showed higher relative abundances in the healthy control group, and evaluated the ratios of these genera.

**Figure 4:**
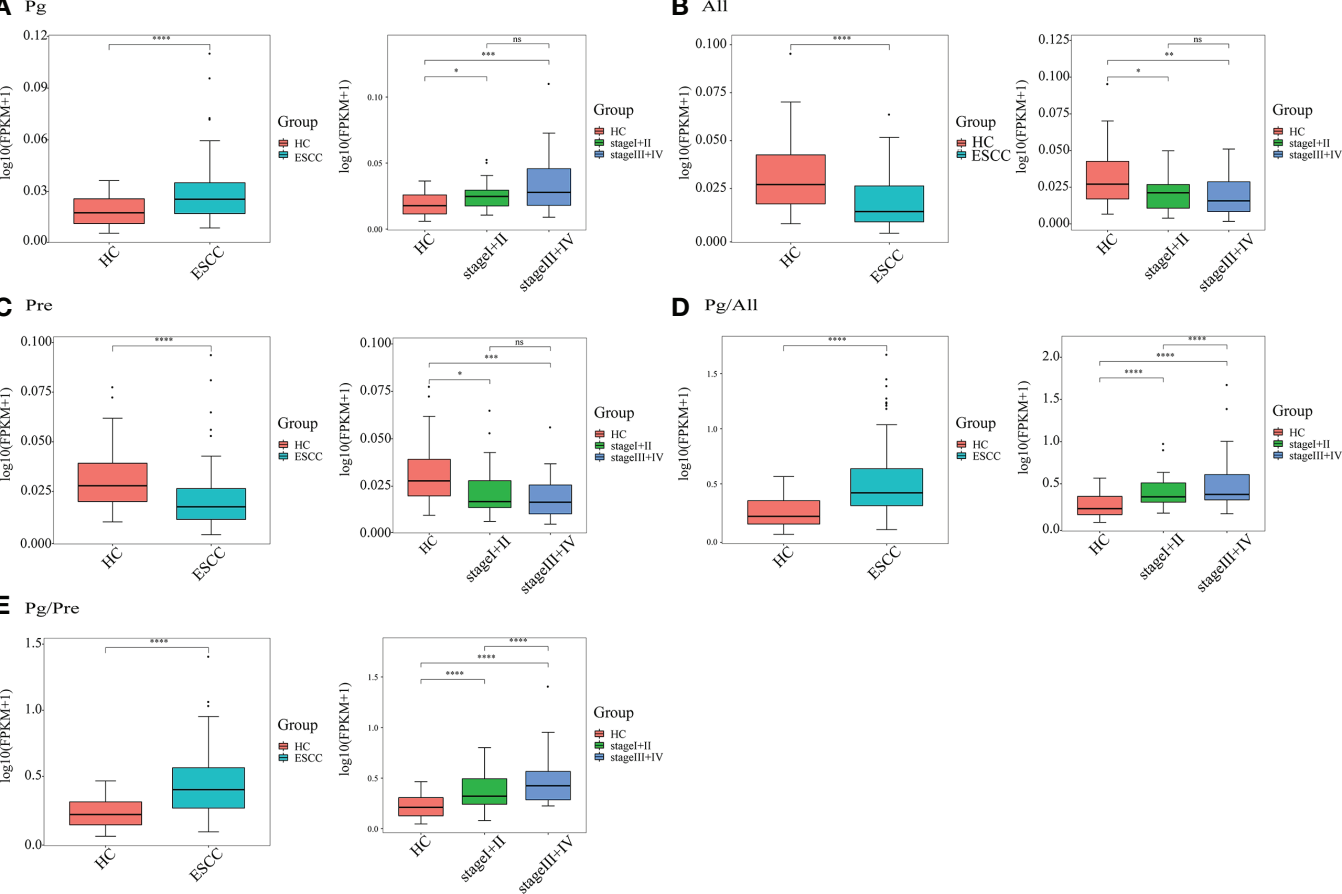
Quantitative detection of saliva microbial genera in the ESCC and healthy control (HC) groups. The relative salivary abundances of **(A)** *Porphyromonas* (*Pg*), **(B)** *Alloprevotella* (*All*), and **(C)** *Prevotella* (*Pre*), as well as the ratios of **(D)** *Pg/All* and **(E)** *Pg/Pre* in all 140 study participants, including 90 ESCC patients and 50 HCs, and in 53 ESCC cases stratified by stage (I+II, n=21; III+IV, n=32) and 50 HCs. *P < 0.01, **P < 0.001, ***P < 0.0001, ****P < 0.00001 and ns, not significant.

As shown in **Figure 4**, the relative abundance of *Pg* in ESCC patients (n=90) was significantly higher than that in healthy controls (n=50, *P*<0.0001), while the relative abundances of *All* and *Pre* in ESCC patients were significantly lower than those in healthy controls (*P*<0.0001). Notably, the abundance of *Pg* in saliva increased significantly with increasing tumor stage (*P*<0.0001). In addition, the ratio of *Pg* to *All* (*Pg/All*) in ESCC patient saliva was significantly higher than that in healthy control saliva (*P*<0.0001). The ratio of *Pg* to *Pre* (*Pg/Pre*) in ESCC patient saliva also was significantly higher than that in healthy control saliva (*P*<0.0001). With the differences in the *Pg/All* and *Pg/Pre* ratios were highly significant between ESCC patients and healthy controls, they also were significantly different according to the stage of ESCC. These results show the potential ability of bacterial ratios to identify ESCC and facilitate its early diagnosis.

### Diagnostic value of salivary microbial ratios Pg/All and Pg/Pre for ESCC

**Figure 5:**
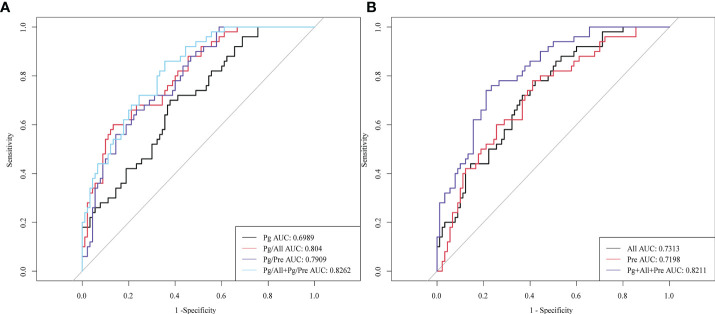
Diagnostic performance of salivary microbial ratios *Pg/All* and *Pg/Pre* compared with *Pg*, *All*, and *Pre* individually or in combination. ROC curves for the ability to distinguish ESCC patients from HCs for **(A)** *Pg*, the ratio of *Pg/All*, the ratio of *Pg/Pre*, and the combinations of *Pg*+*All* and *Pg*+*Pre*; and for **(B)** *All*, *Pre*, and the combination of *Pg* + *Pre* + *All*.

To determine whether the two bacterial ratios *Pg/All* and *Pg/Pre* have diagnostic value for ESCC, we plotted ROC curves to determine the cut off values for distinguishing ESCC patients from healthy controls. The area under the ROC curve (AUC) values for ESCC diagnosis of *Pg*,*All*, and *Pre* individually were 0.6989, 0.7313, and 0.7198, respectively, whereas those for the *Pg/All* and *Pg/Pre* ratios were 0.8040 and 0.7909, respectively (**Figure 5**). Moreover, compared with *Pg* alone, *Pg/All* and *Pg/Pre* had higher sensitivity values (86.7% and 93.3% vs 62.2%), slightly lower specificity values (60.0% and 62.3% vs 70.0%), higher positive predictive values (79.6% and 81.5% vs 78.9%), and higher negative predictive values (71.4% and 83.8% vs 50.7%). In addition, the combinations of *Pg* +*All*+ *Pre* did not show significantly improved diagnostic value (AUC,0.8211, respectively), positive predictive value (85.9%), negative predictive value (62.9%), specificity (78.9%) or sensitivity (74.0%) compared with *Pg/All*+*Pg/Pre* (AUC, 0.826; positive predictive value: 81.1%; negative predictive value: 71.1%; specificity: 64.4%; sensitivity: 86.0%). These results suggest that the combinations of *Pg/All* and *Pg/Pre* in ratio form provide additional value for the diagnosis of ESCC compared with *Pg* alone.

### Value of salivary microbial ratios Pg/All and Pg/Pre for detecting early ESCC

**Figure 6:**
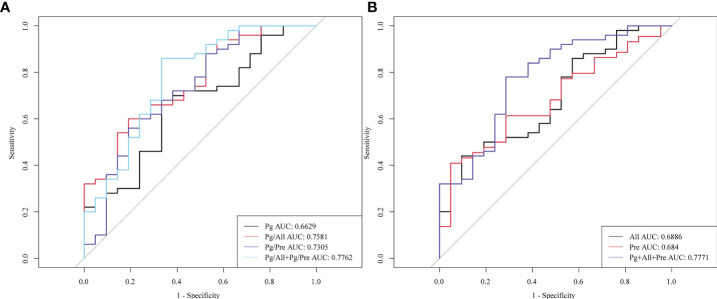
Diagnostic performance of the salivary microbial ratios *Pg/All* and *Pg/Pre* alone or in combination for detection of early ESCC. ROC curves for the diagnostic strength to distinguish stage I+ II ESCC patients from HCs for **(A)** *Pg*, the ratio of *Pg/All*, the ratio of *Pg/Pre*, and the combination of *Pg* + *Pre* + *All*; and for **(B)** *All*, *Pre*, and both the *Pg/All* and *Pg/Pre* ratios combined.

Next, we evaluated the performance of the identified salivary microbiological indicators in the detection of early esophageal cancer in 21 patients with early ESCC (Phase I, n=5; phase II, n=16). As shown in **Figure 6**, the AUC for early ESCC detection for *Pg* alone was only 0.6629, whereas these AUC values for Pg/All and Pg/Pre were 0.7581 and 0.7305, respectively. The *Pg/All* ratio showed a higher sensitivity than *Pg* alone (86.4% vs 68.2%), with a specificity of 54.0%. In addition, the AUC value for the two ratios, *Pg/All* and *Pg/Pre*,in combination reached 0.7762, with a specificity of 86% and no change in sensitivity (68.2%). These results confirm the potential of the *Pg/All* and *Pg/Pre* ratios as biomarkers for improved diagnostic specificity for early ESCC detection and show that the combination of these two ratios offered even better diagnostic value for screening early ESCC.

## Discussion

The present study investigated the associations between alterations in oral microbiota and the presence of ESCC based on 16S rRNA gene sequences identified in human saliva. We found no significant difference in the overall oral microbiota diversity or composition between ESCC patients and healthy control participants, which is consistent with the findings of a recent study that enrolled more than 1000 healthy adults (Lamont et al., 2018). At the genus level, the oral microbiome among individuals is known to be relatively stable, and more than 99% of individuals have 11 core genera, which together account for about 78% of the total relative abundance of bacterial communities (Lamont et al., 2018). The core genera consist of *Streptococcus*;*Veillonella*, *Gemella* and *Granulicatella* of Firmicutes; *Neisseria* and *Haemophilus* of Proteus; *Pre* of Bacteroides; *Actinomycetes* of Actinomycetales; and fusobacteria and actinomycetes, some of which have a relatively low abundance (average relative abundance <2%). However, several species have been found to be increased in the saliva of ESCC patients, including *Leptotrichia*, *Porphyromonas*, *Streptococcus*, *Rothia*, *Lactobacillus*, and *Peptostreptococcus*. These bacteria may be related to the occurrence and development of tumors; for exampls, *Streptococcus* enrichment is a major factor in the case of oral squamous cell carcinoma (Sasaki et al., 2005). However, previous studies (Hajishengallis and Lamont, 2014) have shown that *Porphyromonas gingivalis* is more closely related to esophageal cancer, and thus, we chose *P. gingivalis* as our main research object. In the present study, we adopted a new form of microbial indicator, the ratio of strains and identified the ratios of *Pg/Pre* and *Pg/All* as providing greater diagnostic value for ESCC than any of the individual indicators.

*Pg* is the bacterial genus most closely linked to periodontitis and is known to play a key role in the development of periodontitis by invading epithelial cells or interfering with host immunity (Nearing et al., 2020). A previous study (Hajishengallis and Lamont, 2014) also showed that the detection rate of *P. gingivalis* in ESCC tumor tissues was higher than that in adjacent normal and healthy control mucosa. In addition, the presence of *P. gingivalis* is known to be associated with ESCC lymph node metastasis and shortened survival time (Hajishengallis and Lamont, 2014). In the present study, we also observed an association between an increased relative abundance of *Pg* in saliva and the presence of ESCC. Moreover, for early ESCC, we also observed a significant increase in the relative abundance of *Pg* compared with the control group. Finally,with advancing ESCC stage, we observed enrichment of *Pg* in patients’ saliva, which further indicates that *Pg* may be related to the occurrence and development of ESCC, a finding not previously reported by other studies. However, the sensitivity for using *Pg* alone to detect ESCC was not high, and the AUC was only 0.699, which indicates that the prediction efficiency of a single bacterial type for ESCC is insufficient.

Currently, regular screening and early diagnosis of ESCC are the keys to controlling the incidence rate and mortality burden of ESCC worldwide. A single specific bacterial biomarker does not seem to offer good diagnostic performance though, as also observed by Guo et al., who reported an AUC for a single nucleic acid in bacteria of only 0.635 for colon cancer (Guo et al., 2018). It is well established that microorganisms in a microenvironment interact often with each other, and that such interactions contribute to diseases. As the second largest flora bank in the human body, oral microbiota offer a promising biomarker source for the occurrence of diseases. In our previous research (Wei et al., 2022), we analyzed the differences of the flora in saliva by 16s rRNA sequencing and found that *P. gingivalis* and *Streptococcus salivarius* were enriched in the saliva of ESCC patients compared with healthy controls. Additionally, the abundance of *Fusobacterium nucleatum* was increased, but the predictive performance of a single bacterium for ESCC was low. Therefore, we selected common bacteria in human oral cavity (*Pre*, *Pseudoprevotella*) as our internal references and applied the ratio method to identify biomarkers that showed improved performance on ROC curve analysis. Our study showed that *Pg* was enriched and *All* and *Pre* were reduced in patients with ESCC, whereas the ratio of the enriched to reduced microbial biomarkers *Pg/All* showed an excellent sensitivity of 93.3% and specificity of 63.3%. Thus, the use of the ratio method in this study identified a biomarker with greatly improved diagnostic efficacy. Importantly, for patients with early ESCC, the identified microbial ratio also provided greater diagnostic accuracy. The discovery of this ratio for ESCC detection is a key advantage of this study compared with previous studies.

The present study has some limitations. First the DNA content in saliva is influenced by eating habits and saliva collection conditions, and we were unable to collect oral mucosa or supragingival tissue samples for use as internal references, which may affect the composition and diversity of oral microbiota. However, in the process of selecting the control group, we attempted to select healthy people with similar eating habits and oral hygiene. Therefore, we expect that the observed inter-group differences remain valid. In addition, due to the case-control study design, our results cannot determine whether the reduction in oral microbial richness leads to ESCC or whether the cancer state affects the oral microecology. It has been speculated that extensive oral lesions in the cancer group may alter the oral microbiota, but further research to test this theory is required.

In conclusion, the present study provides evidence that *Pg* may be associated with an increased likelihood of esophageal cancer, whereas *All* and *Pre* may be associated with a reduced risk of ESCC. The ratios of two microbial biomarkers, *Pg/All* and *Pg/Pre*, offered greater diagnostic efficacy for ESCC than any individual microbial biomarker. Importantly, the combination of both the *Pg/All* and *Pg/Pre* ratios provided an even higher sensitivity for the detection of early ESCC. Thus, the combination of *Pg/All* and *Pg/Pre* can be recommended as a valuable, noninvasive screening biomarker for early ESCC that can be popularized in areas with a high incidence of ESCC.

## Data availability statement

The summary result generated in this study are available from the corresponding author on request. The individualgenetic datasets used and/or analyzed during the current study are not publicly available due to Policy.

## Ethics statement

The studies involving human participants were reviewed and approved by the Ethics Committee of Guangdong Provincial People’s Hospital. The patients/participants provided their written informed consent to participate in this study.

## Author contributions

Conceived and designed the experiments: ZL, GQ. Performed the experiments: XC, BX, and JW. Analyzed the data: XC, BX, and JW. Contributed reagents/materials/analysis tools: YC, DY, XLa, XLi, YD, HZ, and WL. Wrote the paper: XC, BX, and ZL. All authors contributed to the article and approved the submitted version.

## Funding

This study was supported by the Scientific Research Project of Guangzhou, Guangdong Province, China [grant number20184010458].

## Conflict of interest

The authors declare that the research was conducted in the absence of any commercial or financial relationships that could be construed as a potential conflict of interest.

## Publisher’s note

All claims expressed in this article are solely those of the authors and do not necessarily represent those of their affiliated organizations, or those of the publisher, the editors and the reviewers. Any product that may be evaluated in this article, or claim that may be made by its manufacturer, is not guaranteed or endorsed by the publisher.
